# AN AGE-FRIENDLY NURSING HOME PROJECT ECHO IN RURAL COMMUNITIES

**DOI:** 10.1093/geroni/igac059.2743

**Published:** 2022-12-20

**Authors:** Lee Jennings, Teri Round, Diana Sturdevant, Keith Kleszynski

**Affiliations:** University of Oklahoma Health Sciences Center, Oklahoma City, Oklahoma, United States; University of Oklahoma Health Sciences Center, Oklahoma City, Oklahoma, United States; University of Oklahoma Health Sciences Center, Oklahoma City, Oklahoma, United States; University of Oklahoma Health Sciences Center, Oklahoma City, Oklahoma, United States

## Abstract

We launched an Age-Friendly Nursing Home Project ECHO in October 2021 focused on the 4Ms (mobility, medications, mentation, what matters most) with emphasis on dementia and QI in long-term care. One-hour virtual sessions included a short expert presentation and case discussion, were offered twice weekly, and recordings were posted online. Topics included person-centered care, advance care planning, LGBTQIA+ care, fall prevention, medication reduction, dementia resident activities, oral health, skin integrity, staff retention, COVID-19, PDSA cycles, root cause analysis, team huddles, among others. 235 individuals from 80 nursing homes, 19 ALFs, 6 continuing care communities, and 10 VA-affiliated sites in 5 states (OK, AR, KS, MO, CO) participated in the first four 6-week ECHO series; 27% attended ≥2 series. Most attendees were nursing home administrators (46%), directors of nursing (20%), nursing assistants (12%), or activity directors (11%). Most were female (91%) and worked in rural settings (77%). 237 attended an additional COVID-19 update session. 152 participants (65%) completed an evaluation. 91% rated the program as valuable (score≥8/10); 99% would recommend to others; 57% discussed topics with colleagues; 59% reviewed materials after sessions; 25% implemented new QI processes; and 25% made a change to resident care. Suggestions for future sessions included: active shooter training, antibiotic stewardship, team building, staff-resident communication, dementia training for non-clinical staff, weight loss, capacity determination, and family caregiver support. An age-friendly nursing home Project ECHO was well-received by health professionals in long-term care. ECHO can successfully expand the reach of training in long-term care, especially in rural areas.

